# Beyond Membrane Potential: Exploiting Signal Complexity in Genetically Encoded Voltage Indicators

**DOI:** 10.3390/s26113616

**Published:** 2026-06-05

**Authors:** Nazarii Frankiv, Haeun Lee, Bradley J. Baker

**Affiliations:** 1 Brain Science Institute, Korea Institute of Science and Technology, Seoul 02792, Republic of Korea; frankiv@kist.re.kr (N.F.); haeun.lee@kist.re.kr (H.L.); 2 Division of Bio-Medical Science and Technology, KIST School, University of Science and Technology, Seoul 02792, Republic of Korea

**Keywords:** genetically encoded voltage indicators, optical voltage imaging, ΔF/F, composite optical signals, fluorescence signal interpretation, membrane physiology

## Abstract

Genetically encoded voltage indicators (GEVIs) have long promised optical access to membrane potential, yet their adoption has lagged significantly behind genetically encoded calcium indicators. A central but underappreciated reason is that the metrics used to evaluate and compare GEVIs—fractional fluorescence change (ΔF/F), kinetics, and signal-to-noise ratio—rest on an assumption that is frequently violated: that GEVI fluorescence reflects a single underlying process. In this perspective, we argue that GEVI signals are composite optical measurements, arising from the superposition of voltage-dependent fluorescence, intracellular and nonresponsive signal, background, and contributions from neighboring cells. Under these conditions, ΔF/F is not a measure of sensor sensitivity but a contrast metric whose value depends on baseline fluorescence composition, optical sampling, and imaging configuration. This reinterpretation has two key consequences. First, it explains a substantial source of variability in GEVI performance that is currently attributed to noise or experimental inconsistency. Second, and more importantly, it reveals that the complexity of GEVI signals is not a limitation to be minimized but a resource to be exploited. By resolving composite signal components, GEVIs can report multiplexed physiological variables, expose hidden conformational states of voltage-sensing domains, probe membrane organization, and reveal intracellular and intercellular electrical coupling. We propose that realizing the full potential of GEVIs requires treating ΔF/F not as a gold standard for sensor performance, but as one interpretable component of a richer optical measurement whose structure encodes multiple layers of cellular physiology.

## 1. Introduction

1997 saw the introduction of two novel fluorescent biosensors: genetically encoded voltage indicators (GEVIs) [1] and genetically encoded calcium indicators (GECIs) [2]. In the nearly three decades since, the use of GEVIs has lagged significantly behind that of GECIs. While there are multiple reasons for this disparity, perhaps the most influential is ease of use. Because GEVIs report changes in membrane potential [3,4], their fluorescence is restricted to the plasma membrane, limiting the amount of signal that can be collected and placing greater demands on efficient protein trafficking. In addition, membrane-localized fluorescence reflects cell surface area and is distributed across fine neuronal processes, reducing local signal intensity. In contrast, GECIs are distributed throughout the cytoplasm, producing signals dominated by cell volume—particularly the soma—resulting in more robust, spatially homogeneous optical signals.

The temporal dynamics of the two modalities also differ importantly. Because GEVIs report membrane potential directly, voltage imaging requires faster acquisition frame rates to capture the full range of electrical events, which in turn reduces photon collection per frame compared to calcium imaging. Calcium signals, while generally slower due to the indirect nature of the readout—cytoplasmic calcium must accumulate and bind the indicator—can also be fast depending on the volume of the compartment being imaged [5,6]. Beyond timing, the two modalities operate over fundamentally different signal ranges. Intracellular calcium concentration is very low at rest and rises substantially with even a single action potential, meaning calcium imaging effectively operates between two easily differentiated states. Furthermore, GECIs cannot reliably report neuronal inhibition except when baseline firing rates are sufficiently high that a reduction in spiking produces a measurable decrease in calcium signal; in the more common scenario of low or absent baseline activity, hyperpolarization produces no detectable response. Voltage imaging, by contrast, reports membrane potential across a continuous and physiologically meaningful range from hyperpolarized states through resting potential to depolarization providing access to a richer repertoire of electrical signals including inhibition, subthreshold synaptic potentials, and graded responses. This richer repertoire, however, also complicates the signal output, as the fluorescence response of a GEVI must encode a wider range of membrane potential changes, making careful interpretation of the optical signal all the more important.

**Figure 1:**
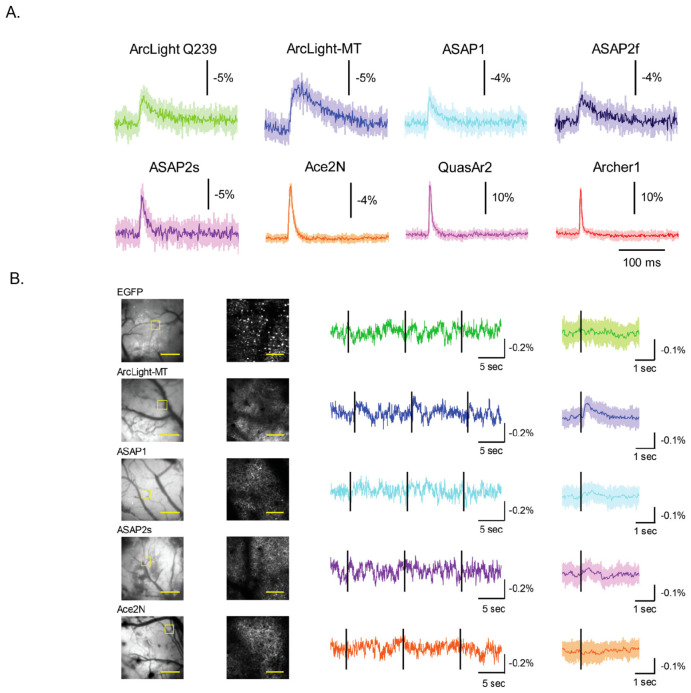
GEVI performance is context-dependent rather than absolute. (**A**) Optical responses to depolarizing voltage steps recorded from cultured hippocampal neurons expressing eight GEVIs representing different voltage-sensing mechanisms. Under in vitro conditions, where individual cells can be selected for optimal membrane expression and regions of interest restricted to plasma membrane-localized fluorescence, all indicators produce measurable voltage-dependent signals, with differences in amplitude, kinetics, and signal composition reflecting their distinct underlying mechanisms. (**B**) Visually evoked optical responses recorded from the same indicators in the primary visual cortex in vivo using one-photon imaging. One-photon images are to the left. Scale bar is 1 mm. Yellow box indicates region used for two-photon image on the right (scale bar is 100 μm) Representative traces of single trial recordings with 10 ms light flashes (vertical bars). Traces on right are the average of visually evoked optical responses over 10 trials. The relative performance of indicators shifts substantially between the two conditions, with ArcLight-MT emerging as the most reliable detector of visually evoked activity despite not exhibiting the largest fractional change in vitro. Rather than reflecting a universal ranking of indicator quality, these results illustrate that the properties that favor performance in one measurement context—signal amplitude, kinetics, brightness, membrane localization—interact differently with the imaging conditions of another. No single metric measured in vitro predicts which indicator will be most informative in a given application. Panels adapted from Bando et al., 2019 [14], under CC BY license.

As a result, GEVI development [7,8] has focused heavily on improving signal amplitude, speed, and signal-to-noise ratio to approach the usability of GECIs. However, these efforts are typically evaluated using metrics such as fractional fluorescence change (ΔF/F) and response kinetics which implicitly assume that GEVI fluorescence reflects a single underlying process. Increasing evidence suggests that this assumption is not always valid [9,10,11,12,13]. Instead, GEVI signals often reflect the integration of multiple photophysical and biological processes whose contributions depend on cellular context, membrane environment, and imaging conditions. Variability arising from this composite signal may contribute to the more limited adoption and practical use of GEVIs compared to genetically encoded calcium indicators (Figure 1) [14].

In this perspective, we examine what follows from treating GEVI fluorescence as a composite optical measurement rather than a single-variable readout. The examples discussed here draw primarily from our own work, as the framework we propose has not yet been systematically applied across GEVI architectures or laboratories. We show that this perspective not only reframes how variability in GEVI recordings should be interpreted, but expands what GEVIs can be used to measure—from membrane potential alone to multiplexed physiological signals, conformational dynamics, membrane organization, and cellular connectivity. Rather than representing limitations to be engineered away, the complexity of GEVI signals can, under the right conditions, reflect the complexity of the biological systems they inhabit.

## 2. Noise, Signal Composition, and the Interpretation of ΔF/F in GEVI Measurements

**Figure 2:**
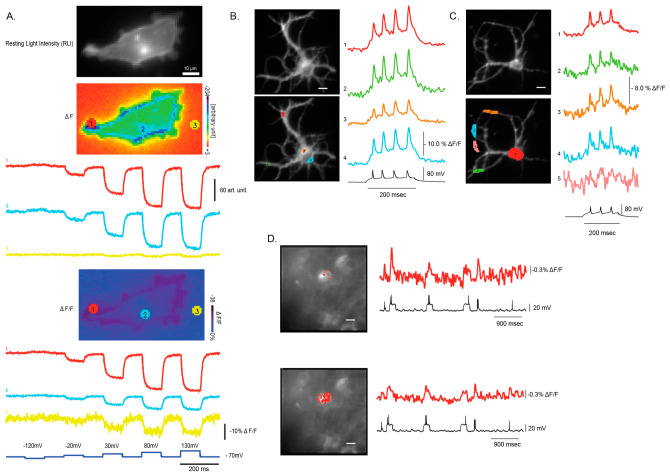
The composite nature of the fluorescence baseline determines the measured ΔF/F independent of the underlying voltage signal. (**A**) Fluorescence responses recorded from three spatially distinct regions of a single HEK 293 cell expressing the GEVI Bongwoori during stepped voltage commands. ROI1 is selected from the plasma membrane and yields the largest fractional signal, as membrane-localized fluorescence with minimal intracellular contribution maximizes the voltage-responsive fraction of the baseline. ROI2 corresponds to a bright intracellular region most likely reflecting Golgi-retained protein that has not trafficked to the outer membrane (Bal et al., 2025 [28]) analogous to VSD internalization which adds to baseline fluorescence without contributing to ΔF, resulting in a markedly suppressed ΔF/F despite a similar absolute fluorescence change. ROI3 is dominated by scattered light with strong background contribution, producing elevated noise and poor signal fidelity. These three regions were chosen deliberately to illustrate how ROI placement decisions often made implicitly determine the quality and interpretability of the measurement. The voltage-dependent signal itself is unchanged across all regions; only the baseline composition differs. Panel adapted from Lee et al., 2016 [10]. (**B**) The same principle operates at the level of subcellular compartments within a single neuron. In a cultured hippocampal neuron expressing Bongwoori-R3, the region of interest containing high internal fluorescence shows nearly 50% reduction in optical response to action potentials compared to membrane-localized regions, despite receiving the same voltage signal. (**C**) Dim regions with low photon counts display degraded signal-to-noise ratios that prevent reliable resolution of action potentials, illustrating that baseline brightness independently limits detectability. (**D**) In an ex vivo retinal preparation, the close proximity of neighboring ganglion cells introduces background fluorescence that cannot be excluded by ROI selection, resulting in an approximately 10-fold reduction in signal-to-noise ratio compared to isolated cultured neurons. Despite this, voltage transients as small as 20 mV remain detectable, illustrating both the challenge and the residual sensitivity of GEVI-based measurements in tissue contexts. Panels (**B**–**D**) adapted from Jung et al., 2025 [18]. Optical traces are color coded to the ROIs.

In optical recordings using GEVIs, the measured fluorescence signal is often treated as a direct readout of membrane potential. However, in practice, the detected signal represents the integration of multiple spatially and mechanistically distinct contributions within the imaging volume [15,16,17]. These include voltage-dependent fluorescence from the plasma membrane [10], intracellular and nonresponsive fluorescence [12], background signal [18], and potential contributions from neighboring cells (Figure 2). As a result, the observed fluorescence cannot be attributed to a single underlying process, but instead reflects a composite optical measurement whose interpretation depends on both biological context and experimental configuration, including illumination geometry, excitation intensity, and acquisition strategy, which can influence the amplitude, kinetics, and signal-to-noise characteristics of voltage-dependent fluorescence responses [19,20,21,22].

Variability in GEVI fluorescence signals is commonly attributed to measurement noise arising from photon statistics, detector limitations, or background fluorescence [17]. While these factors contribute to baseline fluctuations, experimental observations indicate that a substantial portion of this variability reflects unresolved structure within the optical signal itself. A clear illustration of this complexity emerges from optical voltage imaging in neuronal and tissue preparations, where the apparent SNR depends strongly on the spatial definition of the ROI (Figure 2A). Regions with high internal fluorescence exhibit reduced fractional signal changes (Figure 2B), whereas dim regions display increased baseline variability due to photon shot noise (Figure 2C). Expanding the ROI improves photon statistics and reduces apparent noise, but at the cost of attenuating signal amplitude by incorporating nonresponsive or weakly responsive fluorescence (Figure 2D). Thus, variability arises not only from stochastic processes but also from how the measurement integrates heterogeneous spatial signals, making the observed fluorescence inherently dependent on analysis choices.

Although the examples discussed here draw primarily from fluorescent protein–based GEVIs, the composite nature of the fluorescence signal is not restricted to this architecture. Rhodopsin-based indicators, while benefiting from low baseline fluorescence, remain sensitive to illumination conditions and acquisition strategy in ways that can alter the measured signal independently of the underlying voltage change. The specific factors contributing to signal complexity differ across architectures, but in each case the recorded fluorescence potentially reflects more than a single underlying process.

The problem of signal contamination is not unique to GEVI recordings. In calcium imaging, neuropil fluorescence—arising from the dense overlap of axons, dendrites, and glial processes surrounding a somatic region of interest—has long been recognized as a source of signal mixing, and substantial effort has gone into developing correction strategies to address it [23]. In GEVI recordings, an analogous but distinct problem operates within the cell: the fluorescence collected from a single ROI integrates contributions from membrane-localized, voltage-sensitive signal and intracellular, nonresponsive fluorescence that cannot be separated by spatial filtering alone. This within-cell baseline heterogeneity has no direct parallel in calcium imaging, where cytoplasmic distribution of the indicator makes somatic fluorescence relatively uniform. As a result, correction strategies developed for calcium imaging do not translate directly to GEVI data, and the composite nature of the GEVI baseline represents a problem that remains largely unaddressed by existing analytical frameworks.

Additional structure arises in tissue environments, where background fluorescence and the close proximity of labeled cells introduce signal mixing [24,25]. In densely labeled preparations, fluorescence recorded from a single ROI can include contributions from neighboring cells and processes, leading to spatially correlated fluctuations that are often treated as noise [17]. However, as discussed in Section 3.5, such signals can reflect underlying biological interactions including subthreshold events, action potentials, and distal responses mediated through synaptic or electrical coupling, indicating that variability across regions may arise from network-level signal propagation rather than measurement artifact.

The fluorescence signal is further shaped by the superposition of multiple photophysical and physiological processes [9]. ArcLight-derived indicators, for example, exhibit both voltage-dependent and pH-dependent components with distinct spatial localization and kinetics [11,13]. Membrane-associated signals display rapid, reversible responses to voltage, whereas cytoplasmic signals can show slower, persistent changes associated with intracellular acidification [11]—components that may be interpreted as baseline drift or noise if not resolved, but which encode distinct physiological information as discussed in Section 3.

Together, these observations indicate that variability in GEVI recordings cannot be fully described by stochastic noise models alone. Instead, variability reflects a combination of stochastic noise and contributions from spatial averaging, network interactions, and overlapping photophysical processes—all of which are ultimately captured in the metrics used to quantify GEVI performance.

This reinterpretation has direct implications for the use of ΔF/F. The widespread assumption that normalization to baseline fluorescence removes differences in probe expression level, brightness, and imaging conditions is only partially valid. The baseline fluorescence (F) is itself a composite quantity consisting of both voltage-sensitive and voltage-insensitive contributions, and its composition varies substantially across indicators, cells, and experimental conditions [14,20,26]. When a significant fraction of the baseline is nonresponsive, the measured ΔF/F is effectively diluted, underestimating the true voltage-dependent signal. Conversely, indicators with a higher fraction of responsive fluorescence may exhibit larger ΔF/F values despite producing fewer absolute photons. This relationship is further complicated by an asymmetry between brightness and signal fidelity: brighter indicators yield higher photon counts and reduced relative noise, improving detectability even when ΔF/F is modest, whereas dim indicators may display larger ΔF/F values but poorer signal fidelity [26]. Regions with similar absolute fluorescence changes (ΔF) can therefore yield markedly different ΔF/F values depending on baseline intensity, introducing a fundamental asymmetry whereby strong signals may be underestimated and weak or noisy signals overestimated, complicating comparisons across spatial regions, cells, and experimental conditions. Reported ΔF/F values can also depend on excitation conditions and acquisition strategy, further emphasizing that these measurements are not solely intrinsic properties of the indicator [21]. Recent in vivo studies further demonstrate that GEVI recordings can reflect complex interactions between neuronal firing patterns, probe kinetics, and fluorescence-state dynamics, where rapid electrical events may merge or appear distorted within the optical trace [27]. As a result, ΔF/F reflects the composition of the baseline as much as the properties of the indicator itself.

At high magnification, individual cellular compartments such as the plasma membrane can be resolved, allowing ROI selection that minimizes contributions from intracellular or nonresponsive fluorescence, and under these conditions ΔF/F more closely reflects the behavior of the voltage-sensitive component. In contrast, low-magnification imaging—commonly employed for capturing activity across neuronal populations or circuits—reduces spatial resolution such that individual pixels integrate fluorescence from multiple subcellular compartments and, in many cases, multiple cells. This spatial averaging inherently mixes membrane-localized, voltage-sensitive fluorescence with intracellular, background, and potentially nonresponsive signals, such that the measured ΔF/F represents a weighted average of heterogeneous sources rather than a compartment-specific readout. Optical magnification is therefore not merely a technical parameter but a determinant of the effective measurement itself [19]—changes in magnification redefine the physical meaning of the measured fluorescence signal rather than simply altering spatial detail.

Beyond spatial sampling, illumination modality itself can fundamentally alter the measured signal in ways that are independent of the underlying voltage change. For rhodopsin-based FRET-opsin GEVIs, switching from scanless to scanning two-photon illumination dramatically reduces ΔF/F_0_ not because the voltage signal changes, but because high irradiance inherent to scanning suppresses the absolute fluorescence change ΔF while baseline fluorescence F_0_ continues to rise—effectively collapsing the contrast metric independently of sensor or biological properties [26].

In this framework, ΔF/F should be interpreted as a contrast metric, reflecting the relative change in fluorescence against a composite baseline, rather than as a direct measure of intrinsic sensor sensitivity. Meaningful comparison of GEVI performance therefore requires consideration of both ΔF/F and the underlying fluorescence baseline, including brightness, spatial distribution, and the fraction of voltage-responsive signal.

## 3. Beyond Voltage Reporting: Expanding the Scope of GEVI Measurements

### 3.1. Reframing GEVI Signals as Integrative Readouts

The interpretation of GEVI fluorescence as a composite optical measurement has implications beyond signal quantification, extending to how these probes are conceptually used. Traditionally, GEVIs have been treated as reporters of a single variable—membrane potential—with variability in signal amplitude, spatial distribution, or kinetics viewed primarily as limitations of the measurement. However, as discussed in Section 2, the recorded fluorescence signal integrates multiple photophysical and biological processes whose relative contributions depend on cellular context and imaging conditions.

Within this framework, features often regarded as noise or experimental artifact—such as spatial heterogeneity, baseline fluctuations, or secondary signal components—may instead represent structured information embedded within the measurement. Rather than attempting to eliminate these features through averaging or normalization, they can be interrogated to reveal additional aspects of cellular physiology. In this sense, GEVI signals are more appropriately understood as integrative readouts, encoding multiple layers of information related to membrane potential, local environment, and intracellular state.

This shift in perspective expands the functional scope of GEVIs. Instead of serving solely as voltage sensors, GEVIs can be leveraged as probes of coupled physiological processes, where the complexity of the optical signal reflects the underlying complexity of the biological system. The following examples illustrate how this integrative behavior can be exploited to extract information beyond membrane potential.

### 3.2. Multiplexed Physiological Reporting Within a Single Construct

**Figure 3:**
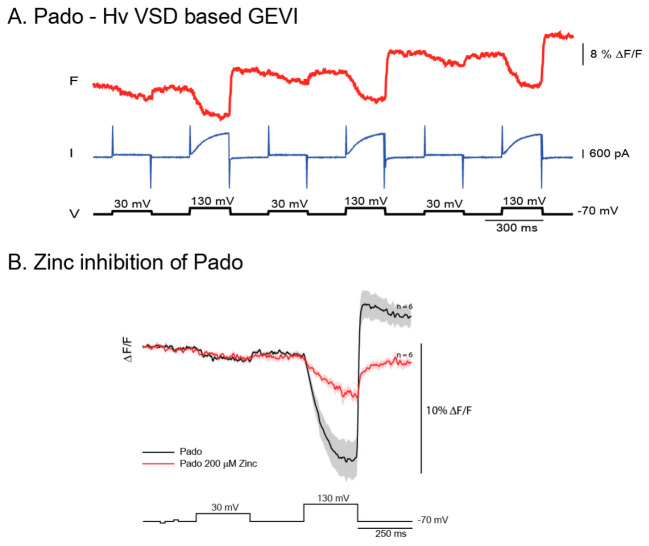
Fluorescence resolves composite and hidden conformational states of voltage-sensing domains inaccessible to electrophysiology. (**A**) The GEVI Pado, which couples the voltage-sensing domain of a proton channel to a pH-sensitive fluorescent protein, exhibits two mechanistically distinct optical components that can be resolved within a single recording. A 100 mV depolarization step produces a voltage-dependent fluorescence change in the absence of ionic current, reporting conformational rearrangements of S4 that do not reach the channel open state. Increasing the depolarization to 200 mV produces a larger optical signal accompanied by a voltage-dependent proton current and a slow rise in baseline fluorescence reflecting intracellular alkalization. The recorded signal thus integrates both voltage-dependent conformational dynamics and a coupled physiological process—intracellular pH change—within a single fluorescence measurement. (**B**) Inhibition of S4 movement by extracellular zinc prevents the channel from reaching the open state, eliminating the proton current and the associated pH-dependent baseline shift. Critically, a voltage-dependent fluorescence change persists in the absence of current, confirming that the optical signal arising from closed-state S4 conformational rearrangements is mechanistically distinct from channel opening. Together, panels (**A**,**B**) demonstrate that GEVI fluorescence provides access to conformational information that is systematically unavailable from electrophysiological recordings alone—resolving closed-state transitions of the voltage-sensing domain that produce no measurable ionic current. Panels adapted from Kang and Baker, 2016 [11], under CC BY license.

A clear example of the integrative nature of GEVI signals is provided by constructs that couple voltage sensitivity with additional physiological processes. The GEVI Pado utilizes the voltage-sensing domain of a proton channel, enabling simultaneous modulation of membrane potential and intracellular pH within a single cell [11] (Figure 3). In this system, voltage-dependent and pH-dependent fluorescence changes are mechanistically distinct yet temporally coupled, producing composite optical signals that integrate both electrical and chemical dynamics.

Analysis of Pado fluorescence reveals that distinct components of the optical signal can be resolved based on their kinetics, amplitude, and spatial distribution, indicating that they arise from separable underlying processes. Membrane-localized fluorescence changes track voltage-dependent conformational rearrangements of the voltage-sensing domain, whereas slower components reflect intracellular pH changes associated with proton flux through the channel. Notably, the pH-dependent fluorescence changes correlate with voltage-dependent current, consistent with proton conduction, while additional fluorescence transitions at subthreshold membrane potentials occur in the absence of measurable current. These observations indicate that the GEVI can resolve multiple closed states of the voltage-sensing domain that are not distinguished by electrophysiological measurements alone, similar to voltage-clamp fluorometry measurements [29]. As a result, the recorded fluorescence signal reports changes in membrane potential while also resolving distinct conformational states of the voltage-sensing domain associated with closed and open channel configurations.

These results illustrate that GEVI fluorescence can encode multiple overlapping processes within a single construct. Rather than representing a limitation, this multiplexed signaling behavior provides an opportunity to probe the interplay between electrical activity, intracellular signaling, and protein conformational dynamics. Exploiting this capability will require approaches that move beyond single-metric analysis to resolve the individual components contributing to the composite signal.

### 3.3. Resolving Hidden Conformational and Photophysical States

Fluorescence readouts from many GEVIs—particularly those based on fluorescent protein electrostatics—provide access to conformational dynamics that are not directly observable through electrophysiological measurements alone. Whereas electrical recordings primarily report ionic current and therefore emphasize transitions associated with channel opening, GEVI fluorescence can detect voltage-dependent structural rearrangements that occur across multiple conducting and non-conducting states (Figure 3). Moreover, voltage-driven perturbation of steady-state fluorescence can unmask novel conformational states of both the voltage-sensing domain and the fluorescent protein chromophore—states that are inaccessible or averaged out under resting conditions, and that electrophysiological recordings cannot resolve.

This capability arises from the coupling between the voltage-sensing domain and the fluorescent protein chromophore. Voltage-dependent conformational changes reshape the local electrostatic environment within the fluorescent protein, modulating chromophore configurations and giving rise to distinct fluorescence outputs [9,30]. The extent to which these multiple states can be resolved depends on the design of the GEVI, with fluorescent protein–based indicators exhibiting particularly rich multi-state behavior due to their sensitivity to local electrostatic changes [9,31,32,33]. Because these photophysical responses can differ in amplitude, kinetics, and spectral properties, they provide multiple observables through which underlying conformational states can be distinguished. This interpretation is consistent with insights from voltage-clamp fluorometry studies, where fluorescence signals often precede or diverge from ionic current, indicating that optical readouts can reflect multiple underlying conformational states rather than a single functional transition [29].

Consistent with this view, GEVI signals can exhibit multi-component responses, complex kinetics, and state-dependent behaviors that are not readily explained by a single conformational transition. Rather than reflecting measurement noise or experimental variability, these features can arise from the selective engagement of multiple underlying states whose contributions vary with voltage, membrane environment, and protein configuration [9,11,30,34] (Figure 3). In contrast, indicators based on alternative mechanisms may report voltage through more direct or single-dominant transitions, resulting in simpler fluorescence responses [35,36,37,38,39].

Accordingly, the fluorescent protein can be viewed as an electrostatically sensitive reporter that maps conformational changes in the voltage-sensing domain onto multiple distinct photophysical states. The set of accessible chromophore configurations is determined by the electrostatic architecture of the protein [9,12,30,31], while the trajectory through these states is influenced by voltage-dependent structural rearrangements. This coupling enables the optical resolution of conformational landscapes that are otherwise averaged or obscured in electrical recordings. It should be noted that while supraphysiological voltage steps were used to reveal these multi-component behaviors, the secondary fluorescence components described here are also observable, albeit in milder form, at +100 mV depolarizations. The use of larger voltage steps served to amplify and clarify the underlying mechanistic structure rather than to imply that these phenomena only occur outside the physiological range.

Importantly, this expanded sensitivity is not limited to the detection of additional states, but also provides insight into how these states are organized and accessed. Differences in fluorescence amplitude, kinetics, and spatial distribution can reflect variations in state occupancy, transition pathways, and local membrane context. As a result, GEVI measurements can be used not only to detect multiple conformational states, but also to infer aspects of the underlying energy landscape governing voltage-dependent behavior.

These observations indicate that, in these systems, GEVI fluorescence provides access to a richer description of voltage-sensitive protein dynamics than is available from electrophysiological measurements alone. By resolving both conducting and non-conducting states, as well as multiple photophysical transitions of the chromophore, GEVIs function as probes of coupled conformational and electrostatic processes. Leveraging this capability will require analytical approaches that integrate multiple features of the optical signal to reconstruct the underlying state dynamics.

### 3.4. Sensitivity to Membrane Organization and Local Environment

Genetically encoded voltage indicators are embedded within the plasma membrane, positioning them to directly sense not only membrane potential but also the local physicochemical environment of the membrane itself. As a result, GEVI fluorescence can exhibit spatial heterogeneity that reflects variations in membrane organization, curvature, and local electrostatic context [9,11].

**Figure 4:**
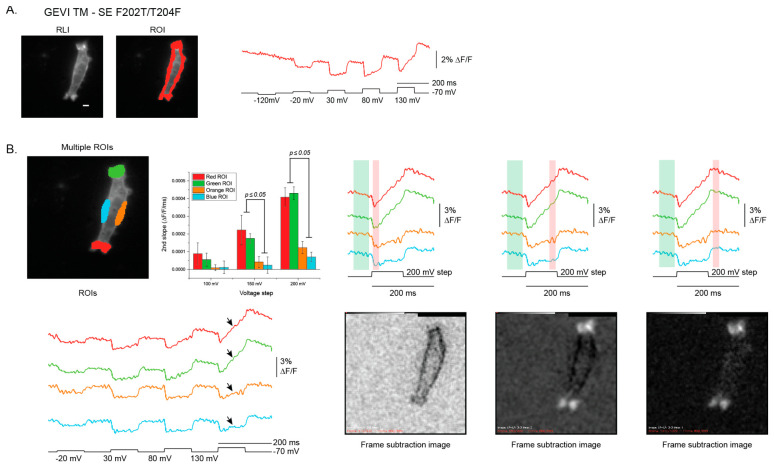
GEVI fluorescence reports local membrane organization: sensitivity to membrane curvature as a reporter of subcellular context. (**A**) Voltage-dependent fluorescence response of an HEK 293 cell expressing the GEVI construct TM-SE F202T/T204F, in which inversion of the external polarity flanking threonine 203 in the FP domain permits increased chromophore flexibility during voltage-sensing domain activation. Depolarizing steps of increasing amplitude produce a progressively enhanced secondary fluorescence component, reflecting voltage-dependent conformational dynamics within the fluorescent protein domain that are distinct from the primary voltage-dependent signal. Scale bar represents 10 μm. (**B**) Within the same cell, the secondary fluorescence component varies systematically across the plasma membrane as a function of local membrane geometry. Regions of interest selected from more highly curved membrane areas exhibit a consistently steeper secondary component compared to regions of flatter membrane, as quantified by linear fitting of the fluorescence trace during sustained depolarization. Frame subtraction images (red shaded frames subtracted from green reference frames) confirm that while all membrane regions initially dim in response to depolarization, curved regions subsequently develop an opposing brightening signal that drives the secondary component (arrows). This spatial heterogeneity is not observed in constructs lacking conformational flexibility at the chromophore, indicating that curvature sensitivity emerges specifically under permissive conformational states of the fluorescent protein domain. Together, these results demonstrate that GEVI fluorescence can encode information about local membrane organization and geometry in addition to voltage, and that spatial variability in GEVI signals should not be assumed to reflect differences in membrane potential alone. Panels adapted from Leong, Shin, Frankiv et al., 2025 [9].

Experimental observations using fluorescent protein–based GEVIs have demonstrated that voltage-dependent fluorescence responses can vary systematically across the cell membrane. In particular, regions of increased membrane curvature can exhibit distinct fluorescence behavior compared to flatter membrane areas, with differences in signal amplitude and secondary components that depend on excitation conditions and local geometry [9] (Figure 4). Although the curvature-dependent differences in signal amplitude and secondary components are most clearly resolved at supraphysiological voltage steps, these effects are also present at +100 mV depolarizations, indicating that they are relevant within voltage ranges accessible in experimental contexts, even if less pronounced. These effects indicate that the fluorescence response is influenced not only by membrane potential, but also by the structural and electrostatic properties of the membrane environment.

Within the framework described in Section 3.3, such spatial variation arises from differences in how local membrane geometry and composition modulate both the voltage-sensing domain and, subsequently, the fluorescent protein chromophore. Changes in membrane curvature can alter lipid packing, membrane tension, and the orientation or conformational flexibility of membrane-associated proteins. These factors, in turn, influence the electrostatic landscape experienced by the chromophore, biasing the relative occupancy of different photophysical states and modifying the observed fluorescence response.

Importantly, these spatial effects are not limited to curvature alone, but may also reflect broader aspects of membrane organization, including lipid composition, microdomain structure, and protein–protein interactions. As a result, GEVI fluorescence can act as a sensitive reporter of local membrane context, with variations in signal reflecting underlying heterogeneity in membrane properties rather than uniform voltage changes.

This sensitivity has important implications for both interpretation and application. From an analytical perspective, spatial variability in GEVI signals should not be assumed to reflect differences in membrane potential alone, but may instead encode information about local membrane structure and composition. From a functional perspective, this behavior provides an opportunity to use GEVIs as probes of membrane physiology, enabling the study of how electrical activity interacts with membrane organization at subcellular resolution.

Taken together, these observations extend the role of GEVIs beyond voltage sensing, positioning them when signals are present and interpretable as reporters of coupled electrical and membrane-dependent processes. It should be noted that not all GEVI fluorescence carries meaningful voltage information; distinguishing genuine signal from non-reporting fluorescence remains an important practical consideration in the design and interpretation of GEVI experiments. In combination with their sensitivity to conformational dynamics (Section 3.3) and multiplexed physiological signals (Section 3.2), this spatial responsiveness highlights the potential of GEVIs to provide integrated measurements of cellular state across multiple levels of organization.

### 3.5. Reporting Cellular Connectivity and Intracellular Electrical Coupling

Genetically encoded voltage indicators can also reveal electrical interactions that extend beyond the plasma membrane of individual cells. Because GEVI fluorescence reflects the integration of signals within the imaging volume, optical recordings can capture activity arising from neighboring cells, subcellular compartments, and coupled membrane systems. As a result, fluorescence changes observed in a given region may reflect not only local membrane potential, but also electrical connectivity within and between cells.

In multicellular and tissue contexts, this behavior is evident in the detection of signals in regions that are not directly stimulated [11,18]. Fluorescence changes recorded from neighboring cells or distal regions can arise from synaptic transmission, ephaptic interactions, or direct electrical coupling through gap junctions. In optical imaging workflows, such signals are often treated as mixed or background fluorescence during ROI-based analysis, particularly when spatial averaging and signal separation approaches are applied. However, in the context of GEVI measurements, these signals may instead reflect biologically meaningful propagation of electrical activity across cellular networks.

This distinction has implications for how analysis strategies developed for calcium imaging are applied to GEVI data. Background subtraction and spatial filtering approaches are commonly used to isolate signals of interest from neuropil or non-specific fluorescence, and considerable effort has gone into developing robust implementations of these methods for calcium imaging data [23]. While appropriate for many calcium imaging contexts, these approaches carry an additional risk when applied to GEVI recordings. Neuropil calcium signals, though often treated as background, have been shown to carry meaningful information correlated with network activity [23], and direct comparisons between GECI and GEVI population signals have demonstrated that the two modalities report fundamentally different aspects of the spatial propagation of neural activity [40]. Because voltage signals are distributed across the membrane and can propagate through cellular networks, fluorescence recorded outside a defined ROI may remain correlated with the activity of interest rather than representing non-specific background. Under these conditions, background subtraction could remove physiologically meaningful signal components, and its application to GEVI data warrants careful consideration of the preparation and imaging context.

**Figure 5:**
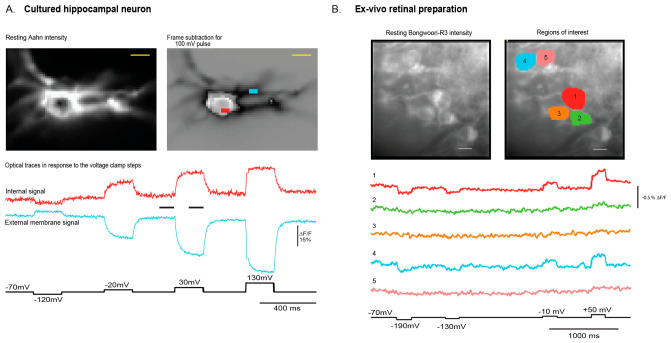
GEVI fluorescence reveals electrical connectivity across spatial scales: from intracellular membranes to intercellular networks. (**A**) Simultaneous optical recording of plasma membrane and internal membrane voltage signals in a cultured hippocampal neuron expressing the GEVI Aahn, which traffics to both the plasma membrane and intracellular membranes. During depolarizing voltage steps, the plasma membrane signal decreases in fluorescence (blue trace), while a spatially distinct internal signal increases with opposite polarity (red trace). Frame subtraction images confirm that the reversed signal is localized to the cell interior, overlapping with endoplasmic reticulum marker distribution, while the plasma membrane signal predominates at the cell periphery. The similar time courses of the two signals indicate that voltage changes at the plasma membrane are rapidly transmitted to internal membrane compartments, revealing an electrical coupling between the plasma membrane and the ER that is not accessible through electrophysiological recordings alone. Scale bar is 10 μm. (**B**) Intercellular electrical coupling detected by GEVI fluorescence in retinal ganglion cells expressing Bongwoori-R3. A centrally located cell under whole-cell voltage clamp is subjected to a series of depolarizing and hyperpolarizing voltage steps. A spatially distinct, non-stimulated cell in the upper left of the field of view exhibits correlated fluorescence changes, including responses to hyperpolarizing steps of the clamped cell. The ability of the non-stimulated cell to follow hyperpolarization—a response that cannot be mediated by chemical synaptic transmission—identifies the presence of a direct electrical synapse between the two cells. Scale bar is 10 μm. Together, panels (**A**,**B**) illustrate that GEVI fluorescence can report electrical interactions across spatial scales ranging from intracellular organelle membranes to intercellular networks, and that signals which might otherwise be dismissed as background or non-specific fluorescence can encode biologically meaningful connectivity information. Panel (**A**) adapted from Sepehri Rad, Cohen, Braubach and Baker [41], under CC BY license. Panel (**B**) adapted from Jung et al., 2025 [18]. Optical traces are color coded to ROIs.

In addition to intercellular coupling, GEVI signals can also provide insight into electrical interactions between the plasma membrane and intracellular membranes [41,42] (Figure 5). Many intracellular organelles, including the endoplasmic reticulum and mitochondria, maintain membrane potentials and are functionally coupled to the plasma membrane through ionic fluxes and signaling pathways. Fluorescence signals arising from intracellularly localized or partially mislocalized GEVIs, or from regions where optical resolution cannot fully separate membrane compartments, may therefore contain contributions from these internal membrane systems.

Importantly, the interpretation of such signals depends critically on optical resolution. At low spatial resolution, fluorescence from plasma and intracellular membranes is spatially averaged, making it difficult to distinguish compartment-specific contributions and increasing the likelihood that intracellular signals are interpreted as background or noise. In contrast, higher-resolution imaging that resolves subcellular structure enables the separation of these components, allowing intracellular fluorescence changes to be analyzed as distinct signals rather than mixed contributions. In this context, optical resolution defines not only spatial detail, but the ability to assign physiological meaning to the measured signal.

In vivo applications further illustrate the expanded utility of GEVIs beyond conventional voltage recording. For example, imaging with the GEVI Marina has been used to detect membrane depolarization in response to endogenous electric fields during collective cell migration, linking optical voltage signals to tissue-level behavior [43]. Such studies demonstrate that GEVI measurements can be used to probe how electrical signals are generated and interpreted within complex biological environments, rather than serving solely as proxies for electrophysiological measurements.

From this perspective, apparent background fluorescence or secondary signal components may, under appropriate imaging conditions, reflect electrical coupling between plasma and intracellular membranes. For example, voltage-dependent fluorescence changes observed in regions lacking clear plasma membrane localization may indicate the propagation of electrical or electrochemical signals into internal compartments, revealing pathways of intracellular connectivity that are not accessible through traditional electrophysiological measurements.

These results demonstrate that GEVI fluorescence can report not only local membrane potential, but also electrical connectivity across spatial scales ranging from subcellular compartments to multicellular networks. In this sense, features that might otherwise be dismissed as noise or signal contamination can instead provide insight into the structure and dynamics of electrically coupled systems.

### 3.6. Optical Sensitivity to Protein–Protein Interactions and Molecular Constraints

In addition to reporting voltage-dependent conformational changes and membrane-associated processes, GEVI fluorescence can be influenced by molecular interactions that constrain protein behavior within the membrane environment. Initial evidence for this comes from our own work examining the effects of membrane trafficking motifs on GEVI performance [44]. The introduction of trafficking sequences derived from ion channels improves plasma membrane expression but also alters the kinetics and voltage dependence of the fluorescence response. These changes cannot be explained by improved localization alone. Critically, inserting a flexible spacer between the fluorescent protein and the trafficking motif restores both kinetics and voltage dependence while preserving the improvement in signal amplitude, consistent with a model in which the trafficking motif restricts conformational dynamics of the voltage-sensing domain, with increased linker flexibility partially relieving this effect, though direct structural evidence for this mechanism remains to be established.

A similar result was seen for the GEVI ArcLight containing a different trafficking motif [45] illustrating a broader principle: that GEVI fluorescence amplitude, kinetics, and voltage dependence can encode information about the molecular context in which the sensor operates, not just its intrinsic properties. Whether analogous interaction-dependent effects are present in other GEVI architectures remains an open question, but one that optical measurements—through systematic comparison of spacer lengths, trafficking motifs, and membrane environments—are well positioned to address.

### 3.7. Design and Analysis Implications for GEVI Measurements

The reinterpretation of GEVI fluorescence as a composite, context-dependent signal has direct implications for both the design of new indicators and the analysis of optical voltage data. Traditional optimization strategies have largely focused on maximizing fractional fluorescence change (ΔF/F), brightness, and kinetics. While these parameters remain important, the framework developed here suggests that GEVI performance depends on the interplay between indicator properties, membrane context, and imaging conditions.

From a design perspective, these considerations highlight the importance of controlling not only the magnitude of the fluorescence response, but also the composition of the baseline signal. Indicators that minimize nonresponsive fluorescence through improved membrane targeting, reduced intracellular accumulation, or enhanced contrast between responsive and nonresponsive states will yield more interpretable signals under a wider range of imaging conditions. At the same time, the presence of multiple photophysical states, particularly in fluorescent protein-based GEVIs, suggests an opportunity to engineer probes that selectively access or separate distinct fluorescence components, enabling more detailed resolution of underlying conformational dynamics. Even within a fixed FRET-opsin architecture, substituting the fluorophore alone can produce unexpected changes in irradiance sensitivity, spectral properties, and kinetics that require new optimization of the full construct [26].

The sensitivity of GEVI signals to membrane organization and local electrostatic environment further suggests that indicator design can be tuned to probe specific aspects of membrane physiology. Rather than treating environmental sensitivity as a source of variability, it may be possible to exploit these effects to generate indicators that report on membrane curvature, lipid composition, or protein interactions in addition to voltage. Similarly, constructs that couple voltage sensing to additional physiological processes, as illustrated by Pado, demonstrate that multiplexed reporting within a single protein can provide access to coordinated cellular dynamics that would otherwise require multiple probes.

Equally important are the implications for data analysis. Many current analysis approaches are adapted from calcium imaging and rely on spatial averaging, background subtraction, and single-metric quantification of signal amplitude [17]. As discussed in Section 3.5, these assumptions may not hold for GEVI measurements, where signals are distributed across the membrane and can exhibit spatial and temporal correlations arising from cellular and network-level interactions. Analytical strategies that treat non-local or secondary signals as noise may therefore obscure meaningful structure within the data.

Instead, analysis of GEVI signals may benefit from approaches that explicitly account for signal composition, including separation of spatial components, identification of multiple kinetic phases, and integration of fluorescence amplitude with baseline intensity and spatial distribution. Incorporating imaging parameters—such as magnification, resolution, and excitation conditions—into the interpretation of GEVI data will also be essential, as these factors directly influence the effective measurement. In this context, the goal of analysis shifts from isolating a single “clean” signal to reconstructing the underlying physiological processes contributing to the observed fluorescence.

Promising directions vary with recording context. For single-cell or high-magnification recordings, where subcellular resolution permits compartment-specific ROI analysis, explicitly separating membrane-localized from intracellular fluorescence offers a straightforward first step toward isolating the voltage-sensitive signal fraction. One practical approach to achieving this separation is frame subtraction, in which fluorescence frames acquired during depolarization are subtracted from baseline frames. This isolates pixels that exhibit genuine voltage-dependent fluorescence changes, effectively identifying voltage-responsive membrane regions while excluding nonresponsive background. As illustrated in Figure 4 and Figure 5, frame subtraction can reveal spatial heterogeneity in the optical response that is not apparent from averaged fluorescence traces alone, and provides a practical basis for informed ROI selection prior to quantitative analysis. Multi-exponential fitting of fluorescence transients can further resolve contributions from multiple photophysical states, and paired reporting of ΔF alongside absolute baseline fluorescence intensity allows post-hoc assessment of baseline composition. For population or low-magnification recordings, where spatial averaging across compartments and cells is unavoidable, spectral or kinetic unmixing approaches that decompose composite fluorescence signals into separable components based on their distinct temporal signatures may provide a more appropriate framework. These approaches are not specific to any single GEVI architecture and could be applied retrospectively to existing datasets to assess the degree of signal mixing in prior recordings.

Together, these considerations suggest that future development of GEVIs should move beyond the optimization of isolated performance metrics toward the design of probes and analytical frameworks that explicitly leverage the composite and information-rich nature of the fluorescence signal. By integrating indicator design with measurement context and data analysis, it may be possible to fully realize the potential of GEVIs as probes of coupled electrical, biochemical, and structural dynamics in living systems.

## 4. Conclusions

Genetically encoded voltage indicators have traditionally been viewed as optical reporters of membrane potential, with performance evaluated in terms of signal amplitude, kinetics, and brightness. However, as illustrated throughout this perspective, GEVI fluorescence arises from the integration of multiple contributing factors, including protein conformational dynamics, membrane organization, optical sampling, and molecular interactions within the cellular environment. As a result, the measured signal cannot be interpreted as a simple, isolated readout of voltage, but instead reflects a structured and context-dependent measurement of cellular state. The convergence of these findings across distinct GEVI architectures and independent laboratories suggests that they reflect a genuine and generalizable principle rather than construct-specific artifacts: that GEVI fluorescence encodes information about the molecular and membrane context in which the sensor operates, not just its intrinsic voltage-sensing properties. Together, these examples support the view of GEVIs as context-dependent optical reporters whose signals reflect coupled electrical, conformational, and membrane-associated processes. We anticipate future studies will test and extend across additional GEVI architectures and experimental systems.

This perspective reframes variability in GEVI recordings not as an experimental limitation, but as a source of information. Differences in signal amplitude, kinetics, and spatial distribution can encode the influence of multiple underlying processes, including access to distinct conformational states, coupling between physiological variables, and interactions across membranes and cellular networks. When appropriately interpreted, these features provide insight into biological mechanisms that are not accessible through electrophysiological measurements alone.

Realizing this potential will require a shift in both indicator design and data analysis. Rather than optimizing single performance metrics, future efforts should focus on controlling signal composition, enhancing the separability of underlying components, and developing analytical approaches that account for spatial, temporal, and mechanistic complexity. In this view, GEVIs provide a means to interrogate how electrical signals are generated, transformed, and integrated within complex biological systems.

## Data Availability

The data presented in this study are available on request from the corresponding author.

## Abbreviations

The following abbreviations are used in this manuscript:

GEVI	Genetically Encoded Voltage Indicator
GECI	Genetically Encoded Calcium Indicator
ROI	Region of interest
